# Sensitivity Equalization and Dynamic Range Expansion with Multiple Optofluidic Microbubble Resonator Sensors

**DOI:** 10.3390/bios13100911

**Published:** 2023-09-28

**Authors:** Ye Wang, Xuyang Zhao, Liying Liu, Xiang Wu, Lei Xu

**Affiliations:** 1 Key Laboratory of Micro and Nano Photonic Structures (Ministry of Education), Department of Optical Science and Engineering, Shanghai Engineering Research Center of Ultra-Precision Optical Manufacturing, Fudan University, Shanghai 200433, China; 14110720005@fudan.edu.cn (Y.W.); 19110720008@fudan.edu.cn (X.Z.); lyliu@fudan.edu.cn (L.L.); 2 Department of Physics, Fudan University, Shanghai 200433, China

**Keywords:** WGM, optofluidic, biosensing, HIV-1 p24

## Abstract

A novel multi-optofluidic microbubble resonator (OMBR) sensitivity equalization method is presented that equalizes the sensing signal from different OMBRs. The method relies on the fact that the ratio of the wavelength shifts to the bulk refractive index sensitivity (BRIS) does not depend on the physical dimensions of the OMBR. The proof of concept is experimentally validated and the sensing signals from individual OMBRs can be directly compared. Furthermore, a wide dynamic range of sensing with favorable consistency and repeatability is achieved by piecing together signals from 20 OMBRs for HIV-1 p24 antigen detection from 50 fg/mL to 100 ng/mL (2.1 fM to 4.2 nM), indicating significant potential for practical applications, such as in drug screening and disease diagnosis.

## 1. Introduction

Traditional biological detection methods, such as ELISA and Western blot, have limitations in terms of sensitivity and detection limits. They typically rely on the chemical amplification and labeling of analytes to generate detectable signals. These complex chemical amplification and labeling processes involve significant time investments, professional equipment, and personnel for extensive measurements, and may affect the target substances being detected. Moreover, these processes often come with high costs. As a result, these limitations hinder their widespread adoption and practical application. Recently, whispering-gallery-mode (WGM) microcavities have been shown to have favorable characteristics over traditional biological detection methods, such as high sensitivity, high-quality factor, and ultralow detection limit, along with being less costly, less time-consuming, and requiring less specialized equipment and personnel. Thus, WGM biosensors are widely used in biosensing to detect single-virus molecules and nanoparticles [1,2,3,4,5,6,7,8]. Optofluidic WGM microcavities (e.g., microbottles, microbubbles, and microtubes) [9,10,11,12,13,14,15,16,17,18,19] attracted intense attention because of their hollow structures, which separate the coupling light from the liquid environment [12,13,14], therefore, more of the light field distributes inside the liquid core [20,21,22]. This dramatically improves the sensitivity and detection limit of the sensors, reduces the amount of liquid required, and provides microfluidic channels for medium delivery that avoid affecting the target substances being detected [23,24].

However, one of the most concerning problems limiting optical biodetection is that the sensitivity of different microcavity resonators differs. Thus, effective sensing data must come from the same microcavity for a comparison to be conducted. Once the operational life of a microcavity is not sufficient to repeat the experiments multiple times [25,26], a real application is impossible, even with a reference resonator. In the latest applications of WGM biosensors, although the durability of microcavities has been constantly improved [21,22,27], their physical and chemical characteristics cannot remain unchanged over the dozens of chemical treatments and rinses required in repeat experiments.

In this study, a novel effective method for equalizing the sensing signals from different experiments with different microbubble resonators is proposed and experimentally achieved. A new parameter, ΔΛ, defined as the ratio of wavelength shifts to BRIS to measure the sensing signal, is defined. The sensing signals of different concentrations of target molecules from different microbubble resonators, regardless of the sensitivity difference, can be compared directly and a smooth curve can be pieced together. Furthermore, the dynamic detection range is remarkably broadened. A complete specific detection sensing curve of HIV-1 p24 antigens ranging from 50 fg/mL to 100 ng/mL is achieved. Meanwhile this type of biosensor is validated to have desirable biocompatibility [28] and can be easily fabricated and conveniently modified with various silane surface coatings by exploiting established silanol surface chemistries to detect multiple proteins or biological tissues, such as BSA, D-biotin, magnolol, IgG, etc. [22,29]. The satisfactory optical sensing performance and biocompatibility of the multi-OMBR sensing system indicate significant potential for practical application in drug screening and disease diagnosis.

## 2. Principle and Experimental Setup

### 2.1. Equalization of Multi-OMBR Sensing Signal

The physical process of equalizing the sensing signal was as follows: the wavelength shift of the WGM was proportional to the surface density of the detecting molecules *σ*, which can be expressed as [30]:

(1) $$\Delta \lambda =\sigma \alpha_{ex}\frac{2\pi \sqrt{n_{w}^{2}-n_{c}^{2}}}{\varepsilon_{0}\lambda^{2}}\frac{n_{w}}{n_{c}^{2}}\lambda S_{bulk},$$

where $n_{w}$ and $n_{c}$ are the refractive index of the OMBR’s silica wall and liquid core, respectively, $\alpha_{ex}$ is the excess polarizability of the target molecules, and *S_bulk_* is the bulk refractive index sensitivity (BRIS), defined as the ratio of the wavelength shift of the WGM (δλ) and the change in the refractive index (δn), which was expressed as follows:

(2) $$S_{bulk}=\frac{\delta \lambda }{\delta n},$$

where Δ*λ* is proportional to *S_bulk_*. It is known that *S_bulk_* depends on the physical properties of a cavity (size, bubble thickness, etc.); therefore, when detecting the same target, sensing signals from different OMBRs are not equal.

Here, we defined a new parameter to measure the sensing signals, ΔΛ, expressed as:

(3) $$\Delta \Lambda =\sigma \cdot A=\frac{\Delta \lambda }{S_{bulk}},$$

where

(4) $$A=\alpha_{ex}\frac{2\pi \sqrt{n_{w}^{2}-n_{c}^{2}}}{\varepsilon_{0}\lambda^{2}}\frac{n_{w}}{n_{c}^{2}}\lambda .$$

As for different microbubble resonators, given the same molecule density captured through the OMBR inner surface, ΔΛ was the same. In this study, ΔΛ instead of Δ*λ* was used as the sensing parameter.

### 2.2. Materials

The miniature quartz tube was from Polymicro Technologies (Phoenix, AZ, USA). The 3-aminopropyltrimethoxysilane (APTMS) was from Thorlabs (Shanghai, China). The bovine serum albumin (BSA) and phosphate-buffered saline (PBS) were from Sigma-Aldrich (St. Louis, MO, USA). The HIV-1 p24 antigen and antibody were from Qitai Bio-tech (Hangzhou, China). The low-refractive-index MY133 polymer was from MY Polymers (Ness Ziona, Israel). The UV glue was from Thorlabs (Shanghai, China).

### 2.3. Experimental Setup

**Figure 1:**
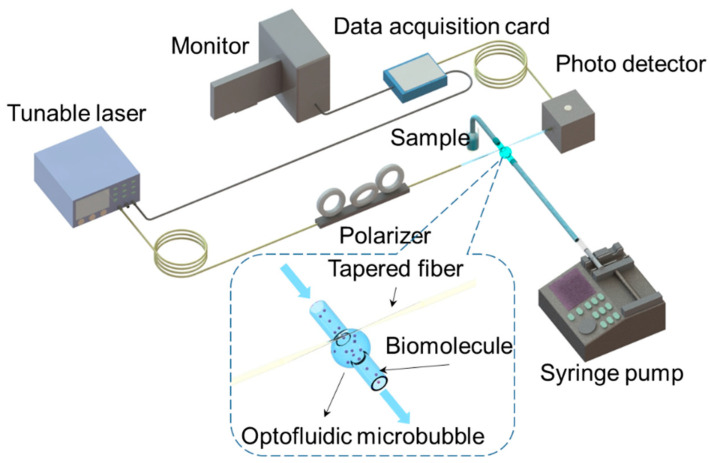
Experimental setup of OMBR sensing system, including optical and microfluidic paths.

Figure 1 schematically shows the experimental setup of the sensing system, which consisted of a tunable laser (TLB6712, 765–781 nm, New Focus, San Diego, CA, USA), a polarization controller (FPC023, Thorlabs, Shanghai, China), a packaged OMBR, a photodetector (APD430A/M, Thorlabs, Newton, NJ, USA), a CCD (Andor iDus DV401A, Belfast, UK), a data acquisition card (PCIE 6351, National Instruments, Austin, TX, USA), a computer, and a syringe pump (Pump 11 Elite Infusion Only Single Syringe 704500, Harvard Apparatus, Holliston, MA, USA). The OMBR was positioned perpendicular to a tapered fiber inside a glass or polymer scaffold caulked with low-refractive-index MY133 polymer around the coupling region and sealed with UV glue. The process of positioning was the same as that described in our earlier work [21].

**Figure 2:**
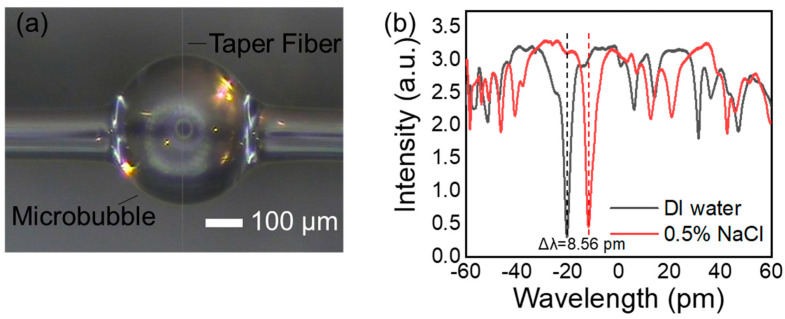
(**a**) Tapered fiber coupled at equator of microbubble. (**b**) Transmission spectra at approximately 780 nm of an OMBR, which was filled with NaCl solution with refractive index difference of 9.5 × 10^−5^. Wavelength shift caused by difference in refractive index was 8.56 pm, corresponding to a BRIS of *S_bulk_* = 9 nm/RIU.

The light from the tunable laser was coupled in-and-out of the microbubble via a tapered fiber, as shown in Figure 2a, and the characteristic parameters were as follows: microbubble outer diameter D = 260 ± 10 μm; wall thickness t = 3 ± 1 μm; diameter of tapered fiber d ≈ 2 μm. Figure 2b shows the transmission spectra from the OMBR when the bubble was filled with DI water and a 0.5% NaCl solution (refractive index difference was Δn = 9.5 × 10^−5^). The quality factor of the cavity was Q = 1.9 × 10^5^, whose wavelength shift caused by the difference in the refractive index was 8.56 pm, and the BRIS was *S_bulk_* = 9 nm/RIU.

### 2.4. Surface Treatment for Specific Detection of HIV-1 p24 Antigen

**Figure 3:**
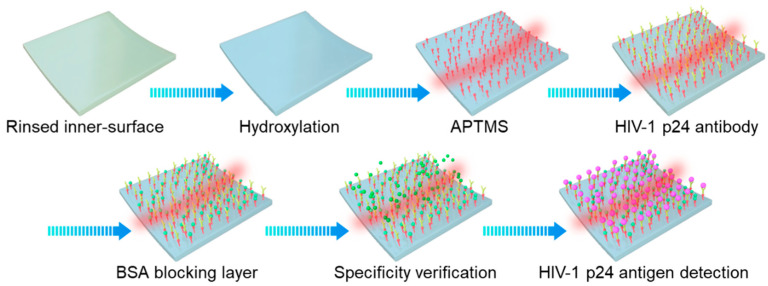
Functionalization of OMBR surface and process of specifically identifying HIV-1 p24 antigen.

The functionalization of the OMBR surface and the process of the specific detection of the HIV-1 p24 antigen are illustrated in Figure 3. The surface of the silica microbubble was covered with a hydroxyl group layer on the back of the atomic binding structure of the SiO_2_, which enabled silyl groups to attach. The inner surface of the OMBR was first hydroxylated with HCl (hydrochloric acid) and H_2_O_2_ (hydrogen peroxide) at 120 °C for 2 h to raise more hydroxyl groups. The volume ratio of 37.5% HCL, H_2_O_2_, and deionized (DI) water was 1:1:5. A layer of APTMS, a silane coupling agent, was first immobilized on the floor of the microbubble at a concentration of 1% in anhydrous ethanol for 2 h; then, the microbubble was washed with anhydrous ethanol and DI water for 10 min each to remove the residual APTMS. Then, the inner surface was functionalized with epoxy groups to immobilize the amino groups of proteins via covalent bonding. To achieve a specific recognition and detection, the strong specificity of the antigens and antibodies was induced. The HIV-1 p24 antibodies, at a concentration of 1 μg/mL, were first attached to the silane coupling agent layer via covalent bonding for 1 h and then washed with 1 × PBS for 30 min. These antibodies were used as the detecting layer to specifically capture the dissociative HIV-1 p24 antigens in solution to be tested. A blocking layer with BSA at 1 mg/mL was introduced for 1 h and then washed with 1 × PBS for 30 min to block vacant binding sites due to the volumetric effects of the antibody macromolecules. The relative molecular mass of the BSA was small enough to ignore the volumetric effect of the colossal antibodies. Before the final detection of the HIV-1 p24 antigens, the BSA solution at the same concentration as the target HIV-1 p24 antigens was introduced into the OMBR at a rate of 2 μL/min for the specificity verification. Then, the HIV-1 p24 antigens were injected into the OMBR, also at a rate of 2 μL/min.

### 2.5. Surface Treatment in Fluorescent Experiments

The inner surface of the OMBR was first hydroxylated with HCl and H_2_O_2_ at 120 °C for 2 h to raise more hydroxyl groups, and this step was only necessary in the approach to recover the OMBR surface environment. The volume ratio was the same as the specific detections of the HIV-1 p24 antigen. Then, a layer of APTES (3-aminopropyltriethoxysilane), a silane coupling agent, was immobilized on the floor of the microbubble at a concentration of 1% in anhydrous ethanol, and any residual was removed with anhydrous ethanol and DI water. After that, the fluorescent-labeled goat antihuman IgG was injected into the OMBR at a rate of 2 μL/min. At last, the OMBR was rinsed with 0.1% HF for 5–10 min and DI water for another 30 min.

## 3. Results and Discussion

### 3.1. Operational Life of OMBRs

The major concern in biosensing detection is that the operational life of the OMBRs is not long enough for many repeated cycles. The wavelength shift under the same density of target molecules becomes progressively less after several detection cycles; eventually, the OMBRs even fail to perceive the antigens because the chemical treatment damages the inner silica surface. To test the durability and deterioration of the chemical decorating and rinsing, fluorescent-labeled goat antihuman IgG antibodies were introduced to track the inner surface modification process. The operation steps were mentioned in Section 2.5. We also attempted to reset the chemical conditions by hydroxylating the inner surface with HCl and H_2_O_2_. Two similar OMBRs were used in the durability test. The parameters of the two OMBRs were D_1_ = 280 ± 10 μm, t_1_ = 5 ± 1 μm and D_2_ = 240 ± 10 μm, t_2_ = 5 ± 1 μm.

**Figure 4:**
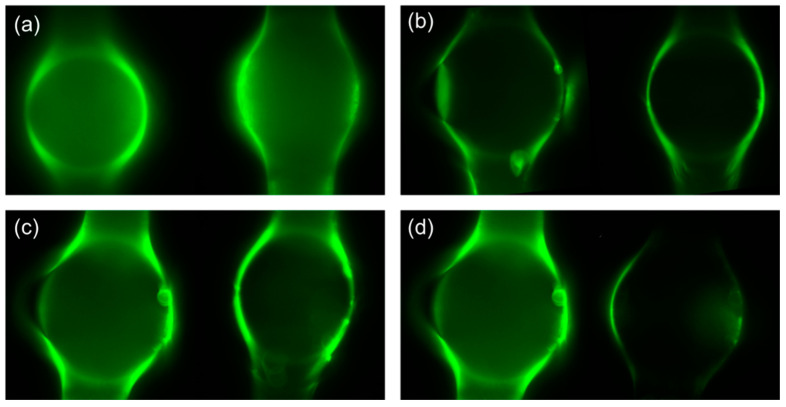
(**a**) Fluorescent images of two newly prepared OMBRs after binding fluorescent-labeled IgG. (**b**) Fluorescent images of antibodies after eight detection cycles with defect points emerging. (**c**) Fluorescent images of antibodies after hydroxylation of surface. (**d**) Fluorescent images before (left) and after (right) rinsing with hydrofluoric acid of left OMBR in (**c**).

Figure 4a shows the fluorescent images of two newly prepared OMBRs after the binding of the fluorescent-labeled IgG. Figure 4b shows the images after decorating and rinsing the OMBRs eight times without hydroxylation to recover the OMBR surface environment. The luminescence was significantly attenuated, and several defect points emerged on the inner surface of the two OMBRs. Figure 4c shows the surface-recovering approach, where the two OMBRs were hydroxylated with HCl and H_2_O_2_ and, again, bound with labeled antibodies. Even though the slightly enhanced luminescence indicated that the binding sites of the silica and silane coupling agent recovered a little, the defects remained and were even worse. Figure 4d shows the fluorescent images before (left) and after (right) rinsing the left OMBR, in Figure 4c, with hydrofluoric acid. The belly of the bottle could not be cleaned completely, and defect points were more likely to emerge in this area where the residue of former decorations and the new chemical decorations mixed into the chaotic substances. In biosensing detection, the equator of the belly is where light couples in and detection reactions take place. There does not appear to be an effective method to stabilize or recover the physical and chemical environment of the OMBRs, of which the operational life is approximately eight times. In recent years, a new surface treatment that uses a glycine-HCl buffer to dissociate the captured antigen–antibody complex was reported [31]. This method makes it possible to detect antigens around eight times higher in one decorating–rinsing cycle. We plan to explore this in our future work to verify its potential to extend the OMBRs’ operational life.

### 3.2. Specific Detection of HIV-1 p24 Antigen

**Figure 5:**
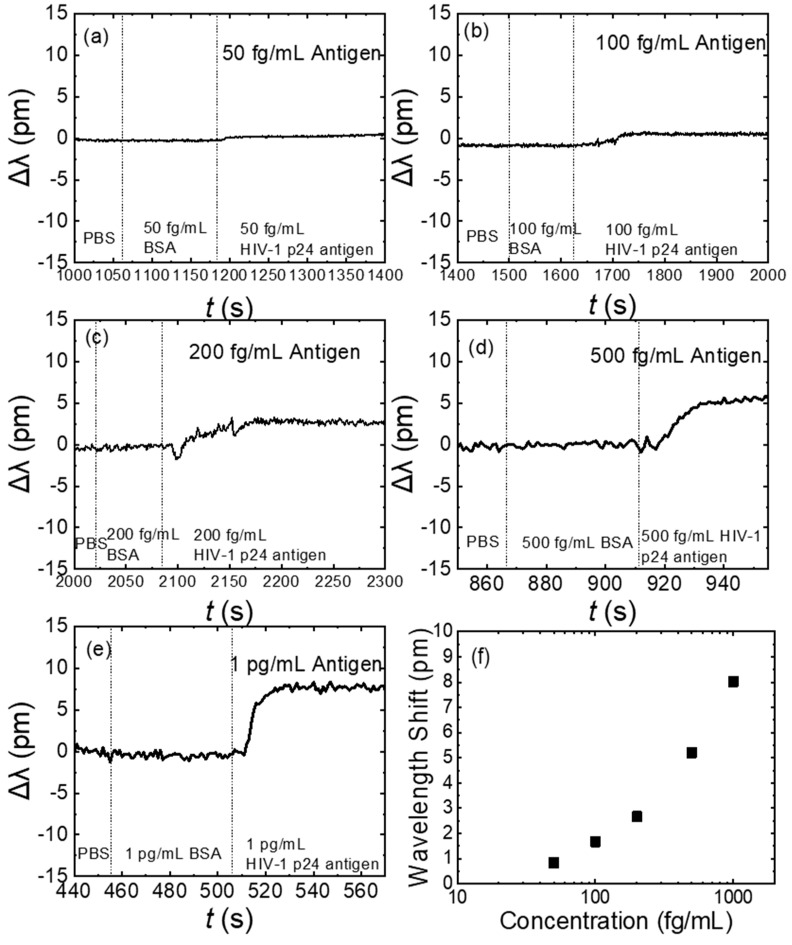
(**a**–**e**) Wavelength shift curves for detection of 50 fg/mL, 100 fg/mL, 200 fg/mL, 500 fg/mL, and 1 pg/mL (2.1 fM, 4.2 fM, 8.3 fM, 20.8 fM, and 41.7 fM) of HIV-1 p24 antigens, respectively. OMBR was soaked in PBS at the beginning to establish a baseline. Before introduction of antigen, BSA at the same concentration was introduced and did not cause any distinguishable shift. (**f**) Sensing curve ranged from 50 fg/mL to 1 pg/mL, showing the relationship between wavelength shift and HIV-1 p24 antigen concentration.

Figure 5a-e illustrate the wavelength shift curves caused by the specific binding of the HIV-1 p24 antibodies and antigens with an OMBR of D = 360 ± 10 μm and t = 3 ± 1 μm. The operation steps were mentioned above. The reaction between the HIV-1 p24 antigens and antibodies was monitored in real time. There was a clear redshift in the spectrum and, ultimately, it became flat again when the antibodies and antigens reached a new dynamic equilibrium. Figure 5f shows the sensing curve, ranging from 50 fg/mL to 1 pg/mL, indicating the relationship between the wavelength shift and the concentration of the HIV-1 p24 antigens. After the HIV-1 p24 antibody connection was confirmed, a high-concentration BSA solution was used for blocking the vacant sites. In Figure 5a–e, before antigen detections, the specificity was tested with a BSA solution with the same concentration as the antigen. At this time, even if a few impurity molecules were to be adsorbed on the surface, their influence on the optical field mode would be much lower than that of the antigen to be detected, and it would not affect the sensing signal of the antigen molecule detection, thus, achieving a high specificity.

However, on one hand, sensing signals from different OMBRs were still not comparable, so the detection relied on the robustness of the nonreplaceable OMBR. On the other hand, due to the limited operational life of the OMBR, the detections with one OMBR for a certain range of concentrations was hard to repeat enough times.

### 3.3. Multi-OMBR Sensitivity Equalization and Dynamic Range Enlargement

In order to verify that a comparison between different microcavities becomes possible when using a new sensing parameter, ΔΛ, we presented a novel method to equalize the sensing signals from multiple OMBR detections. Repeatedly replacing used OMBRs with freshly prepared ones overcame the limited operational life.

**Figure 6:**
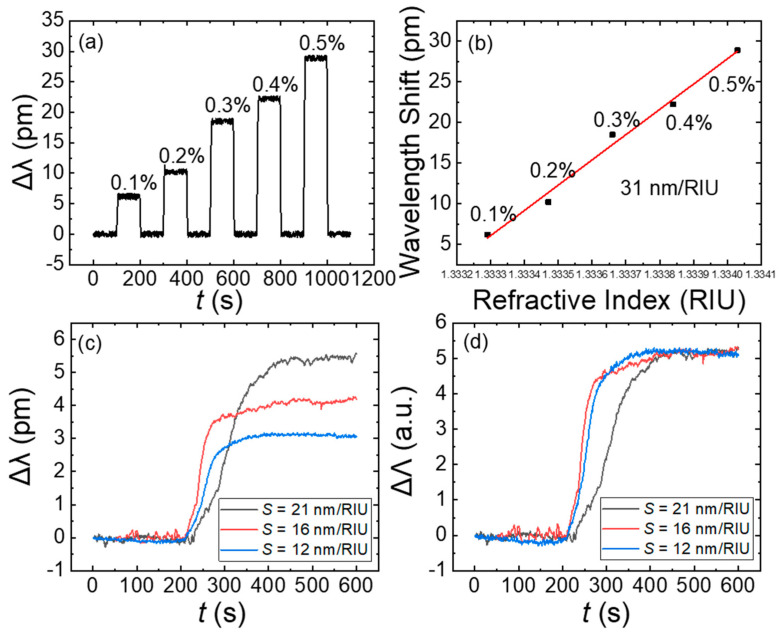
(**a**) Stepped wavelength shifts caused using NaCl solution in linear mass ratio ranging from 0.1% to 0.5%. Before and after NaCl solution was imported into the OMBR, DI water was introduced as a control and as an indication that the system reached equilibrium. (**b**) Linear fit of wavelength shifts of NaCl solutions. Bulk sensitivity *S_bulk_* = 31 nm/RIU. (**c**) Here, 1 pg/mL of HIV-1 p24 antigens was detected using three OMBRs with different BRISs in independent experiments. First, 1 pg/mL of BSA was introduced, which did not cause a distinguishable wavelength shift. Then, 1 pg/mL of antigens was introduced at 200 s, and stable dynamic equilibrium was established after 200 s in each detection. Wavelength shifts varied as a consequence of diverse sensitivities. (**d**) Sensing signals were equalized, and output of three detections was very close regardless of sensitivity differences.

Three OMBRs with D = 360 ± 10 μm and t = 3 ± 1 μm were used to independently detect HIV-1 p24 antigens at a concentration of 1 pg/mL, with BSA as the control group. In order to equalize the sensing signal from the three cavities, the BRIS of each OMBR was measured before the biosensing of the HIV-1 p24 antigen. Figure 6a,b show examples of a measured BRIS. NaCl solutions were prepared at room temperature in a linear mass ratio of 0.1–0.5% with a proportional refraction index of 1.9 × 10^−4^, 3.8 × 10^−4^, 5.7 × 10^−4^, 7.6 × 10^−4^, and 9.5 × 10^−4^ RIU relative to DI water. Before and after the NaCl solution was injected into the OMBR, DI water was introduced as a control. Figure 6a shows the stepped sensorgram of one cavity with parallel stairs, and the drops clearly demonstrated the proportional relationship of the corresponding solution concentration. As shown in Figure 6b, the BRIS was *S_bulk_* = 31 nm/RIU with a standard deviation *σ* = 0.21 pm, and the theoretical detection limit was calculated as DL = 3*σ*/*S_bulk_* = 2.0 × 10^−5^ RIU.

Figure 6c shows the dynamic detected curves of the HIV-1 p24 antigens at a concentration of 1 pg/mL for the three cavities. The difference in sensitivity was due to the submicron level difference in the cavity sizes. The three curves clearly showed that the wavelength shifts had a positive correlation with the BRIS of the OMBR; the one with S = 12 nm/RIU had the smallest redshift of 3.10 pm, the one with S = 16 nm/RIU had a medium redshift of 4.17 pm, and the one with S = 21 nm/RIU had the largest redshift of 5.52 pm. Figure 6d shows the sensing curves after the equalization treatment following Equation (3); Table 1 summarizes the equalization results. The parameter ΔΛ, which is the ratio of the wavelength shift to the BRIS, remained a constant in three independent detections regardless of the sensitivity, which verified that at a certain concentration, the HIV-1 p24 antigen led to the same ΔΛ in the OMBR sensing system.

**Figure 7:**
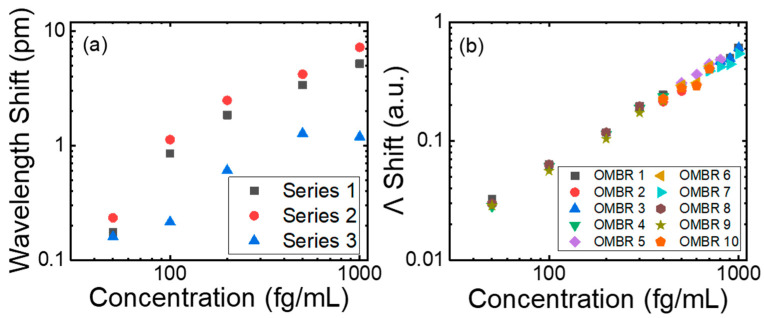
(**a**) Sensing curves of original repeating method for 15 detections with only one OMBR. Detections were stratified into three series, and in each series, HIV-1 p24 antigens at 50 fg/mL, 100 fg/mL, 200 fg/mL, 500 fg/mL, and 1 pg/mL were detected. (**b**) Sensing curve of equalization method with 10 OMBRs to detect HIV-1 p24 antigens ranging from 50 fg/mL to 1 pg/mL at intervals of 100 fg/mL. Each OMBR, noted with the same color and shape, detected antigens three to five times and was then replaced with a new one with similar physical characteristics.

For concentrations below 1 pg/mL, the entire dynamic range could be covered by using one OMBR. However, during the detection, the physical characteristics of the OMBR deteriorated due to modifications and rinsing on the inner surface. Hence, very few OMBRs could be recycled more than 10 times. To compare the stability between the equalization approach and the original repeating approach, two sets of experiments were designed with similar OMBRs of D = 360 ± 10 μm and t = 3 ± 1 μm. As shown in Figure 7a, one OMBR was used for all 15 detections. The detections were stratified into three series; within each series, the detection ranged from 50 fg/mL to 1 pg/mL in five steps, and each series began after the previous one finished. The sensitivity of the OMBR changed significantly in the three series due to the surface treatment. At each concentration, the wavelength shifts in series three shifted significantly from that of series one and two, with a large standard deviation. In addition, it was very difficult to finish the three detection series using the same OMBR. In the other set, shown in Figure 7b, 10 OMBRs were used for the detection from 50 fg/mL to 1 pg/mL with a denser concentration gradient. Each OMBR detected HIV-1 p24 antigens three to five times and was then replaced with another OMBR. As for each concentration, detections were reproduced four or five times. After all detections were completed, the sensing signals were equalized and combined to generate a complete sensing curve. Due to the equalization step eliminating the effect of varying sensitivities, the sensing signals demonstrated remarkable consistency, and the relative deviation for each concentration was much smaller than with the original repeating approach.

Hence, we could directly compare the sensing signals of different OMBRs at different concentrations regardless of their sensitivity differences in each independent experiment. Moreover, the operational life of the OMBR was no longer a factor in the repeatability of the experiments, which led to a much better repeatability than other nonreplaceable WGM resonator biosensing approaches.

**Figure 8:**
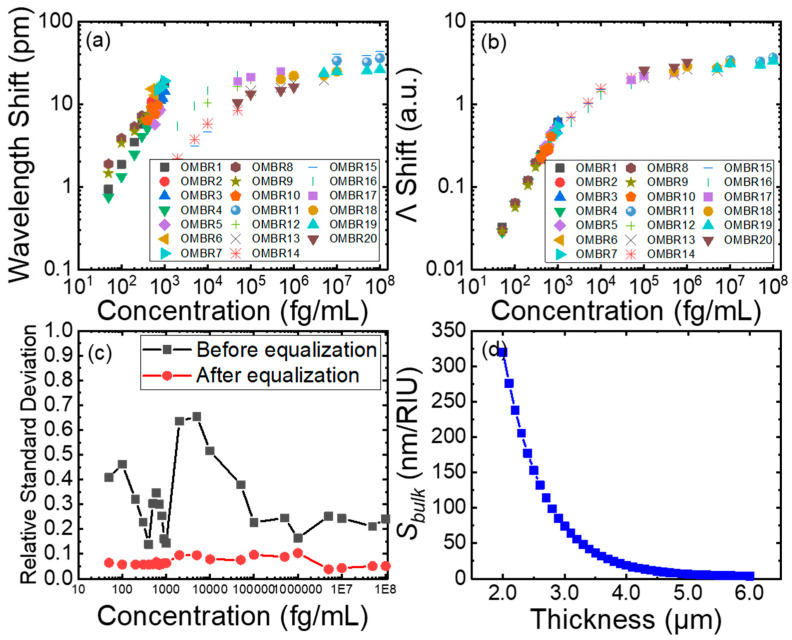
(**a**) Unequalized sensing data ranging from 50 fg/mL to 100 ng/mL in specific detection of HIV-1 p24 antigen. Each OMBR, noted with the same color and shape, detected antigens three to five times and was then replaced with a new one with similar physical characteristics. (**b**) Equalized sensing data. (**c**) Relative standard deviation changes before and after equalization. (**d**) Relationship between *S_bulk_* and wall thickness.

A wide dynamic range of the sensing system is essential in practical applications. However, it is impossible to obtain a wide detecting range (e.g., covering three or more magnitudes of concentration) with one OMBR, because detecting an ultralow concentration and medium concentration requires cavities with different sensitivities. Here, we showed that with the multi-OMBR sensitivity equalization method, the dynamic detection range could be vastly expanded. As shown in Figure 8, a total of 20 OMBRs were used to detect the antigens the HIV-1 p24 antigens at concentrations of 50, 100, 200, 300, 400, 500, 600, 700, 800, and 900 fg/mL; 1, 2, 5, 10, 50, and 100 pg/mL; and 1, 5, 10, 50, and 100 ng/mL, respectively. Each detection was independent. For each specific concentration, detections were reproduced four or five times. All original wavelength shifts are shown in Figure 8a.

All 20 OMBRs had a microbubble outer diameter of approximately 360 ± 5 μm, but with different wall thicknesses. Generally, as shown in Figure 8d, thicker OMBRs generated a lower sensitivity, which was more suitable for the higher concentration range. Therefore, the wall thickness was divided into two types, 3 ± 1 μm for detections below 1 pg/mL and 5 ± 1 μm for those above 1 pg/mL; meanwhile, the diameters were all approximately 360 ± 10 μm. As shown in Figure 8a, the sensitivity of the two types of resonators was very different. Figure 8b shows that, after the equalization, the detecting gap was eliminated and the statistics illustrated a smooth Langmuir sensing curve, which was consistent with the regular adsorption–desorption pattern of concentration gradients [32,33]. Moreover, Figure 8c shows that the average relative standard deviation was remarkably reduced from 31.06% to 6.70%, indicating that the effect of the physical differences in OMBRs was in a satisfying range. In summary, the detecting range was expanded substantially with a consistent low deviation by piecing together sensing signals from different OMBRs.

## 4. Conclusions

In this study, we presented a novel multi-OMBR detecting method to equalize sensing signals from individual microbubble resonators. ΔΛ, defined as the ratio of wavelength shifts to bulk refractive index sensitivity, was used as a new measurement of biosensing signals. This approach overcame the major problem of the limited operational life, and the biosensing dynamic range was remarkably expanded to at least seven orders of magnitude with the multi-OMBR method. The sensing system was optimized to achieve an entire sensing curve of HIV-1 p24 antigens ranging from 50 fg/mL to 100 ng/mL. Moreover, this method had fascinating biocompatibility to promote to various proteins or biological tissues being less costly, less time-consuming, and requiring less specialized equipment and personnel compared to current traditional biological detection methods. In general, the multi-OMBR sensitivity equalization detecting system has practical potential for drug screening and disease diagnosis applications.

## Figures and Tables

**Table 1:** Equalization of wavelength shifts at concentration of 1 pg/mL.

OMBR	*S_bulk_*	Wavelength Shift	Equalized Signal
1	21 nm/RIU	5.52 pm	5.52
2	16 nm/RIU	4.17 pm	5.47
3	12 nm/RIU	3.10 pm	5.43

## Data Availability

Not applicable.

## References

[1] Vollmer F., Braun D., Libchaber A., Khoshsima M., Teraoka I., Arnold S. (2002). Protein detection by optical shift of a resonant microcavity. Appl. Phys. Lett..

[2] Dong C., He L., Xiao Y., Gaddam V., Özdemir S., Han Z., Guo G., Yang L. (2009). Fabrication of high-Q polydimethylsiloxane optical microspheres for thermal sensing. Appl. Phys. Lett..

[3] Sun Y., Fan X. (2011). Optical ring resonators for biochemical and chemical sensing. Anal. Bioanal. Chem..

[4] Arbabi, Goddard L. (2013). Measurements of the refractive indices and thermo-optic coefficients of Si_3_N_4_ and SiO_x_ using microring resonances. Opt. Lett..

[5] Foreman M., Swaim J., Vollmer F. (2015). Whispering gallery mode sensors. Adv. Opt. Photonics.

[6] Yang S., Wang Y., Sun H. (2015). Advances and prospects for whispering gallery mode microcavities. Adv. Opt. Mater..

[7] Zhang M., Liu J., Cheng W., Cheng J., Zheng Z. (2019). A tunable optical Bragg grating filter based on the droplet sagging effect on a superhydrophobic nanopillar array. Sensors.

[8] Zhang M., Cheng W., Zheng Z., Cheng J., Liu J. (2019). Meridian whispering gallery modes sensing in a sessile microdroplet on micro/nanostructured superhydrophobic chip surfaces. Microfluid. Nanofluid..

[9] Li X., Zhang Z., Yin Q., Qiu M., Su Y. Ultra-compact parallel label-free biosensors based on concentric micro-ring resonators in silicon-on-insulator. Proceedings of the Asia Optical Fiber and Optoelectronic 2008 Exposition and Conference.

[10] Zhu H., Dale P.S., Fan X. (2009). Optofluidic ring resonator sensor for sensitive label-free detection of breast cancer antigen CA15-3 in human serum. Proc. SPIE.

[11] Hunt H., Dahmen J., Soteropulos C. (2014). Interfacing whispering gallery mode microresonators for environmental biosensing. Proc. SPIE.

[12] Freeman L., Armani A. (2012). Photobleaching of Cy5 conjugated lipid bilayers determined with optical microresonators. IEEE J. Sel. Top. Quantum Electron..

[13] Zhu J., Özdemir Ş.K., Xiao Y.-F., Li L., He L., Chen D.-R., Yang L. (2010). On-chip single nanoparticle detection and sizing by mode splitting in an ultrahigh-Q microresonator. Nat. Photonics.

[14] Sumetsky M., Dulashko Y., Windeler R.S. (2010). Optical microbubble resonator. Opt. Lett..

[15] Armani A., Kulkarni R., Fraser S., Flagan R., Vahala K. (2007). Label-free, single-molecule detection with optical microcavities. Science.

[16] Berneschi S., Farnesi D., Cosi F., Conti G., Pelli S., Righini G., Soria S. (2011). High Q silica microbubble resonators fabricated by arc discharge. Opt. Lett..

[17] Cosci A., Quercioli F., Farnesi D., Berneschi S., Giannetti A., Cosi F., Barucci A., Conti G., Righini G., Pelli S. (2015). Confocal reflectance microscopy for determination of microbubble resonator thickness. Opt. Express.

[18] Farnesi D., Barucci A., Righini G., Conti G., Soria S. (2015). Generation of hyper-parametric oscillations insilica microbubbles. Opt. Lett..

[19] Wang H., Wu X. (2015). Optical manipulation in optofluidic microbubble resonators. Sci. China Phys. Mech. Astron..

[20] Zhang X., Liu T., Jiang J., Liu K., Yu Z., Chen W., Liu W. (2014). Micro-bubble-based wavelength division multiplex optical fluidic sensing. Advanced Sensor Systems and Applications VI.

[21] Tang T., Wu X., Liu L., Xu L. (2016). Packaged optofluidic microbubble resonators for optical sensing. Appl. Opt..

[22] Zhang X., Liu L., Xu L. (2014). Ultralow sensing limit in optofluidic micro-bottle resonator biosensor by selfreferenced differential-mode detection scheme. Appl. Phys. Lett..

[23] Pablo B. (2016). Optical Microbottle resonators for sensing. Sensors.

[24] Li Z., Zhu C., Guo Z., Wang B., Wu X., Fei Y. (2018). Highly sensitive label-free detection of small molecules with an optofluidic microbubble resonator. Micromachines.

[25] Madugani R., Yang Y., Le V.H., Ward J.M., Chormaic S.N. (2016). Linear laser tuning using a pressuresensitive microbubble resonator. IEEE Photonics Technol. Lett..

[26] Zhi Y., Yu X.-C., Gong Q., Yang L., Xiao Y.-F. (2017). Single nanoparticle detection using optical microcavities. Adv. Mater..

[27] Li M., Wu X., Liu L., Fan X., Xu L. (2013). Self-referencing optofluidic ring resonator sensor for highly sensitive biomolecular detection. Anal. Chem..

[28] Wilson K.A., Finch C.A., Anderson P., Vollmer F., Hickman J.J. (2012). Whispering gallery mode biosensor quantification of fibronectin adsorption kinetics onto alkylsilane monolayers and interpretation of resultant cellular response. Biomaterials.

[29] Guo Z., Lu Q., Zhu C., Wang B., Zhou Y., Wu X. (2019). Ultra-sensitive biomolecular detection by external referencing optofluidic microbubble resonators. Opt. Express.

[30] Hu H., White I.M., Suter J.D., Dale P.S., Fan X. (2007). Analysis of biomolecule detection with optofluidic ring resonator sensors. Opt. Express.

[31] Zhou W., Li K., Wei Y., Hao P., Chi M., Liu Y., Wu Y. (2018). Ultrasensitive label-free optical microfiber coupler biosensor for detection of cardiac troponin I based on interference turning point effect. Biosens. Bioelectron..

[32] Srisa-Art M., Dyson E.C., deMello A.J., Edel J.B. (2008). Monitoring of real-time streptavidin−biotin binding kinetics using droplet microfluidics. Anal. Chem..

[33] Jung L.S., Nelson K.E., Stayton P.S., Campbell C.T. (2000). Binding and dissociation kinetics of wild-type and mutant streptavidins on mixed biotin-containing alkylthiolate monolayers. Langmuir.

